# Note from the editors: 10th European Antibiotic Awareness Day (EAAD) – raising awareness about prudent use of antimicrobials to help curb antimicrobial resistance

**DOI:** 10.2807/1560-7917.ES.2017.22.46.171116-2

**Published:** 2017-11-16

**Authors:** Eurosurveillance editorial team

**Affiliations:** 1 European Centre for Disease Prevention and Control (ECDC), Stockholm, Sweden

**Keywords:** Antibiotic Awareness Day, antimicrobial use, antimicrobial resistance

On 18 November 2017, Europe will mark the 10th European Antibiotic Awareness Day (EAAD) [1]. This European health initiative coordinated by the European Centre for Disease Prevention and Control (ECDC) has brought together many partners and has been one of several European initiatives acknowledging the growing problem of antimicrobial resistance, and recognising that raising awareness about the need for a prudent use of antibiotics in human as well as in veterinary medicine, is an important step in curbing antibiotic resistance.

What started in 2008 as a small initiative that provided a platform for campaigns to promote prudent antibiotic use in the European countries has constantly grown over the years, and in 2017, more than 40 European countries participate. Since 2015, the World Health Organization has been organising a World Antibiotic Awareness Week (WAAW) that is taking place this year from 13 to 19 November with the participation of many countries worldwide [2]. EAAD partners with WAAW, and a number of international organisations and institutes such as the United States Centers for Disease Control and Prevention and the Public Health Agency of Canada join forces on WAAW.

On the occasion of the 10th EAAD, several activities took place in Europe and the ECDC released the latest surveillance data from the European Surveillance of Antimicrobial Consumption Network (ESAC-Net) and the European Antimicrobial Resistance Surveillance Network (EARS-Net).

The good news regarding antimicrobial consumption in Europe is that the consumption of antibiotics (expressed as defined daily doses (DDD) per 1,000 inhabitants per day) in the community i.e. outside hospitals, as well as the consumption in hospitals, did not increase in the European Union (EU) and European Economic Area (EEA) overall and even decreased in several countries, in particular in the community, during the period 2012 to 2016 [3]. Less encouraging, however, is the observation that for specific last-line antimicrobial groups such as carbapenems and polymyxins (mainly colistin), the consumption increased significantly during the same period in more than one third of the countries. 

The good news in the European data on antimicrobial resistance is that the decline in the occurrence of meticillin-resistant *Staphylococcus aureus* (MRSA) at EU/EEA level continued in line with the observations in previous years [4]. However, MRSA levels remain high in several countries. For *Escherichia coli*, combined resistance to three key antimicrobial groups: fluoroquinolones, third-generation cephalosporins and aminoglycosides, increased significantly at EU/EEA level between 2013 and 2016. In contrast, for *Klebsiella pneumoniae*, a small decrease in resistance was observed between 2013 and 2016 for most antimicrobial groups under surveillance. Nevertheless, the fact that resistance to last-line antimicrobial groups such as carbapenems and polymyxins remains high or even increases in some countries is worrisome. Taken together, the newly released data reinforce the need of continued efforts to closely monitor antimicrobial consumption and resistance in Europe in order to contain the development and spread of antimicrobial resistance [5]. 

Antimicrobial resistance has been high on the agenda of *Eurosurveillance* for many years and we have followed the evolution of the EAAD with interest. In this issue, two articles cover different aspects of the surveillance of antimicrobial resistance. Altorf–van der Kuil et al. describe the setup of a comprehensive nationwide laboratory-based surveillance system for antimicrobial resistance in the Netherlands and provide pertinent examples of how data generated by this system have served as evidence for policy making [6]. In addition to describing their present system, the authors outline future developments to improve the system based on the present experiences. In another article, Watier et al. from France, analyse different measurements used to monitor and compare antibiotic consumption between European countries [7]. They conclude that when using the same metrics over time, antibiotic consumption data aggregated and disseminated at European level are useful for assessing temporal trends across Europe and within individual countries and that “*DDD – although imperfect – are the most widely accepted metric for this purpose*”.
